# Suitable Planting Area Prediction for Two *Arnebia* Species: An Analysis Based on Habitat and Phytochemical Suitability

**DOI:** 10.3390/plants14111669

**Published:** 2025-05-30

**Authors:** Yanlin Wang, Shuo Yan, Shanshan Gao, Huanchu Liu, Qi Wang

**Affiliations:** 1 Key Laboratory of Xinjiang Phytomedicine Resource and Utilization of Ministry of Education, Key Laboratory of Oasis Town and Mountain-Basin System Ecology of Xinjiang Production and Construction Corps, College of Life Sciences, Shihezi University, Shihezi 832003, China; 17690564830@163.com (S.Y.); 19b928027@stu.hit.edu.cn (Q.W.); 2 Liaoning Shenyang Urban Ecosystem Observation and Research Station, Shenyang 110164, China; liuhuanchu@126.com; 3 Liaoning Key Laboratory of Urban Integrated Pest Management and Ecological Security, College of Life Science and Engineering, Shenyang University, Shenyang 110044, China; gaoshanliushui0908@163.com; 4 CAS Key Laboratory of Forest Ecology and Silviculture, Institute of Applied Ecology, Chinese Academy of Sciences, Shenyang 110164, China; 5 School of Life Science and Technology, Harbin Institute of Technology, Harbin 150080, China

**Keywords:** *Arnebia* species, medicinal plant, climate change, land use/land cover, suitable planting areas

## Abstract

The distribution of suitable habitats for medicinal plants is affected by climate, soil, land use, and other factors. *Arnebiae Radix*, an important traditional Chinese medicinal resource in Xinjiang, includes *Arnebia euchroma* (Royle) I. M. Johnst. and *Arnebia guttata* Bunge and is at risk of over-exploitation. This study predicted suitable planting areas by integrating habitat and phytochemical suitability using the MaxEnt model and ArcGIS. The AUC values for *A. euchroma* and *A. guttata* were 0.977 and 0.952, with TSS values of 0.829 and 0.725, respectively, validating the high accuracy of the prediction model. Under the current scenario, the areas of suitable habitats for *A. euchroma* and *A. guttata* were 108,914 and 176,445 km^2^, mainly distributed along the main mountains in Xinjiang. Under future climate scenarios, the suitable habitat area of *A. euchroma* increased by 11–18%, except in the ssp126-2090s scenario, while the suitable habitat area of *A. guttata* area decreased by 3–18%. Both species were influenced by land use/land cover and soil available nitrogen content; additionally, *A. euchroma* was affected by the precipitation in the driest month, and *A. guttata* by the mean diurnal range. The content of secondary metabolites was positively correlated with habitat suitability, with soil factors contributing 35.25% to the total secondary metabolite content. Their suitable habitats predominantly occur in grasslands (42–82%). As habitat and phytochemical suitability distributions aligned, the eastern and western sides of the northern Kunlun Mountain Pass emerged as key areas for cultivation. This research can provide a scientific foundation for selecting optimal planting regions for the two *Arnebia* species.

## 1. Introduction

Medicinal plants are plants that contain compounds that are useful for human health and are used to treat or prevent diseases [1,2]; they are an important part of China’s biodiversity. According to the latest census, there are 10,608 species of higher medicinal plants in China, accounting for about 83% of China’s medicinal biological resources [1]. The rapid development of the Chinese medicine market has led to a rapid expansion in the scale of medicinal plant cultivation. However, 70% of commonly used Chinese medicinal materials still rely on wild resources [3]. Due to the growing market demand, most wild medicinal plants, especially species with high commercial value, are facing the threat of over-harvesting. In addition, habitat degradation and loss and climate change also threaten the survival of medicinal plants [4].

The growth and quality of medicinal plants are influenced not only by genetic factors but also by the environment. Suitable planting areas can affect not only the growth of medicinal plants but also the content and composition of their medicinal ingredients [5]. Consequently, predicting suitable planting areas for medicinal plants holds critical significance for both their conservation and quality control in pharmaceutical applications. In recent years, substantial advancements have been achieved in the regionalization of medicinal plant production areas. The integration of remote sensing and geographic information system tools, the expansion of the range of ecological factors, and the improvement of the temporal and spatial resolution of ecological factors have improved the accuracy of regionalization outcomes [6,7].

The advancement of species distribution models (SDMs) has further promoted the regional studies on the origin areas of medicinal plants [8]. SDMs integrate known species distribution data with multiple environmental variables to simulate the geographical distribution of species and their responses to climate change through specific algorithms [9]. SDMs have been used in a variety of species distribution-related studies, including invasive species monitoring [10,11], endangered species conservation [12,13,14], economic species distribution assessment [15,16], and pest control [17,18]. Among various SDMs, the maximum entropy (MaxEnt) model has outstanding advantages, maintaining high accuracy and stability even in the case of partial loss of species data, small sample size, and presence-only data [19]. In addition, MaxEnt also has the advantages of fast operation speed and flexible operation, low storage requirements for computer equipment, and easy visualization of prediction results [20,21,22].

The quality of medicinal plants is fundamentally determined by the content of secondary metabolites. Consequently, researchers have increasingly focused on regional variations in the chemical compositions of these plants. Recent studies have begun to combine the content of secondary metabolites of medicinal plants and habitat suitability to predict suitable planting areas; these are mainly divided into two types of methods. One type of research is to evaluate the content of secondary metabolites and predict the habitat suitability and then evaluate whether the habitat is suitable for the cultivation of medicinal plants [23,24,25,26]. Some studies have found that the content of secondary metabolites of medicinal plants in highly suitable habitats is high [23,24,25], while another study found that the content of secondary metabolites of medicinal plants in less suitable habitats was higher [26]. The other type of research is to establish the relationship between secondary metabolite content and ecological environment factors and to predict suitable planting areas in combination with the prediction of suitable habitats. The emergence of such methods improves the applicability and accuracy of medicinal plant zoning. The growth trend and index components of medicinal plants are very important for the production process of medicinal plants. Therefore, it is effective to combine habitat suitability and phytochemical suitability for the zoning of medicinal plants [5,27,28].

The diverse topography and landforms of Xinjiang have formed unique regional climate characteristics and nurtured rich medicinal plant resources [29]. *Arnebia euchroma* (Royle) I. M. Johnst. and *Arnebia guttata* Bunge are two important medicinal plants, mainly distributed in Xinjiang. The dried roots of these two medicinal plants are *Arnebiae Radix* in the Chinese Pharmacopoeia (2020 version). The two *Arnebiae* species contain rich secondary metabolites, including naphthoquinones, polysaccharides, monoterpene phenols, benzoquinones, and esters, among which naphthoquinone compounds such as shikonin serve as the pharmacological basis for their therapeutic activities. Pharmacological studies have demonstrated that both species exhibit anti-inflammatory, antibacterial, anti-tumor, liver protection, and immunomodulatory effects [30]. Currently, the two Arnebia species are integral components in multiple traditional Chinese medicine formulations for treating diverse clinical conditions [31].

With the growing market demand for the two *Arnebiae* species, their prices have surged [32]. Driven by profit, the wild resources of the two *Arnebiae* species have been illegally mined and excavated without restraint, leading to a steady decline. This over-exploitation contrasts sharply with continuously growing market demand. Currently, all commercial supplies of the two *Arnebia* species rely entirely on wild harvesting, as large-scale commercial cultivation has not yet been established, resulting in severe supply–demand imbalances. Due to the large-scale mining of wild resources, the reserves of the two *Arnebiae* species have dropped sharply. It is difficult to find large areas of them in the wild in Xinjiang, and they are currently in an endangered state [33].

As indispensable traditional Chinese medicine resources, the two *Arnebia* species urgently require standardized cultivation to meet market demand, with the selection of appropriate planting areas being critical. Therefore, the main purposes of this study include (1) predicting the distribution of suitable habitats for the two species in Xinjiang, China; (2) analyzing the main factors influencing their suitable habit and the main factors affecting the content of secondary metabolites; (3) mapping the distribution of phytochemical suitability; and (4) providing a scientific basis for the selection of suitable planting areas for the two species based on habitat and phytochemical suitability. This study will provide guidance for the site selection of standardized planting areas of the two *Arnebiae* species and holds significant importance for conserving their wild populations.

## 2. Results

### 2.1. Model Performance and Key Environmental Variables

For *A. euchroma*, the MaxEnt model achieved an AUC of 0.977 and TSS of 0.829 in predicting suitable habitat distributions, while *A. guttata* yielded AUC and TSS values of 0.952 and 0.725, respectively. Both performance metrics for *A. guttata* were slightly lower than those of *A. euchroma* (Table 1). Overall, the MaxEnt model demonstrated robust performance for both species, indicating highly reliable and accurate prediction outcomes.

**Figure 1:**
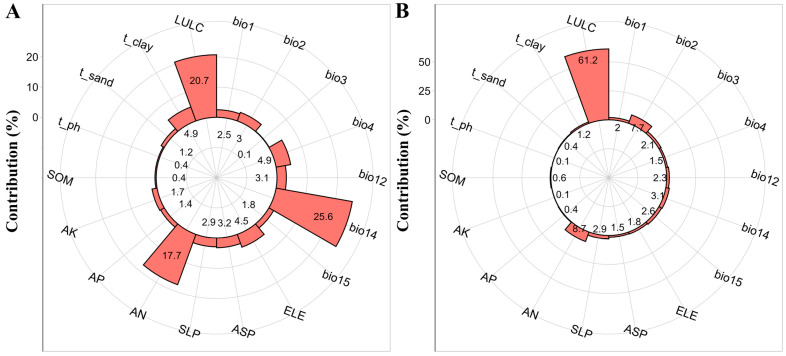
The contribution rates of environmental variables. (**A**) *A. euchroma*. (**B**) *A. guttata*.

The suitable habitat distribution of *A. euchroma* was mainly influenced by the precipitation in the driest month (bio14, 25.6%), LULC (20.7%), and soil available nitrogen (AN, 17.7%), with these three factors collectively accounting for 64.0% of the total contribution. The suitable habitat distribution of *A. guttata* was dominated by LULC (61.2%), AN (8.7%), and mean diurnal range (bio2, 7.7%), with a combined contribution of 78.1%. Notably, both species exhibited significant dependence on LULC and AN as key drivers (Figure 1).

### 2.2. The Suitable Habitat Distribution of Two Medicinal Plants Under the Current Scenario

**Figure 2:**
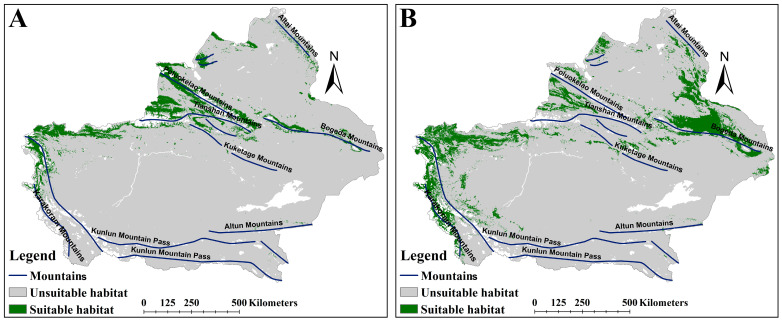
The suitable habitat distribution map of two medicinal plants under the current scenario. (**A**) *A. euchroma*. (**B**) *A. guttata*.

The MSS value was applied to delineate suitable versus unsuitable habitats, with MSS values of 0.18 for *A. euchroma* and 0.16 for *A. guttata*. Both species exhibited shared spatial patterns in suitable habitat distribution, primarily concentrated along major mountain ranges in Xinjiang. Notably, *A. guttata* displayed a more extensive suitable habitat range compared to *A. euchroma* (Figure 2). *A. euchroma* occupied 108,914 km^2^ of suitable habitat (Table 2), spanning the northern Tianshan Mountains, northern and southern slopes of the Poluokelao Mountains, northern slope of the Bogeda Mountains, eastern and western margins of the northernmost Kunlun Mountain Pass, and isolated areas on the western slope of the Altai Mountains (Figure 2A). In contrast, *A. guttata* had a notably larger suitable habitat area of 176,445 km^2^ (Table 2), with a distribution pattern similar to that of *A. euchroma*, but the suitable habitats for *A. guttata* were mainly concentrated on the northern slope of the Bogeda Mountains, the middle part of the Tianshan Mountains and the Poluokelao Mountains, the north slope of the Kuketage Mountains, the eastern and western sides of the northern section of the Kunlun Mountain Pass, and the eastern slope of the Karakoram Mountains (Figure 2B).

Although these habitats were suitable for the growth of both medicinal plants, there were still differences in habitat suitability (Figure S1). Under the current scenario, the areas with higher habitat suitability for *A. euchroma* were mainly located in the northwestern area of the Poluokelao Mountains and the Tianshan Mountains, as well as the west side of the northernmost end of the Kunlun Mountain Pass (Figure S1A). The areas with higher habitat suitability for *A. guttata* were mainly located in the northern area of the Bogeda Mountains, the north of the Tianshan Mountain Pass, the eastern area of the Karakoram Mountains, and other small areas (Figure S1B).

### 2.3. The Suitable Habitat Distribution of Two Medicinal Plants Under the Current Scenario

**Figure 3:**
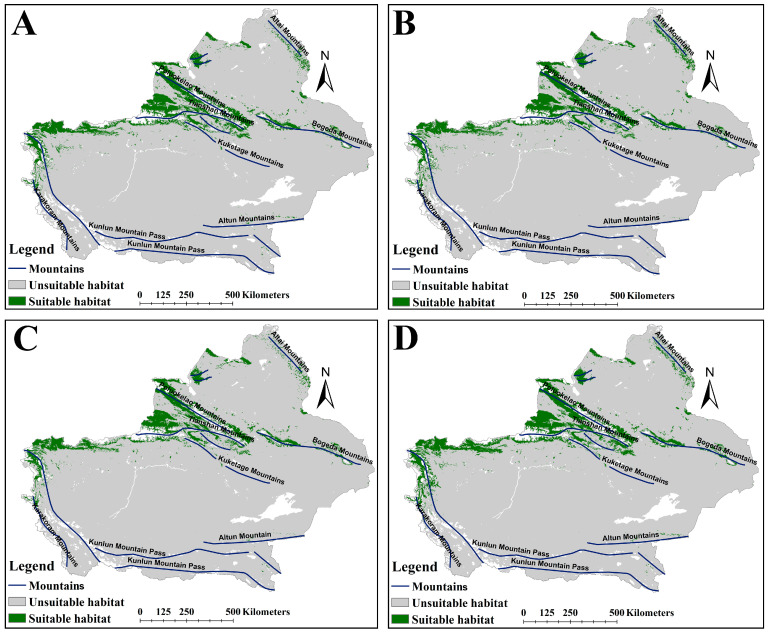
The suitable habitat distribution map of *A. euchroma* under future climate scenarios. (**A**) ssp126-2050s, (**B**) ssp585-2050s, (**C**) ssp126-2090s, (**D**) ssp585-2090s.

**Figure 4:**
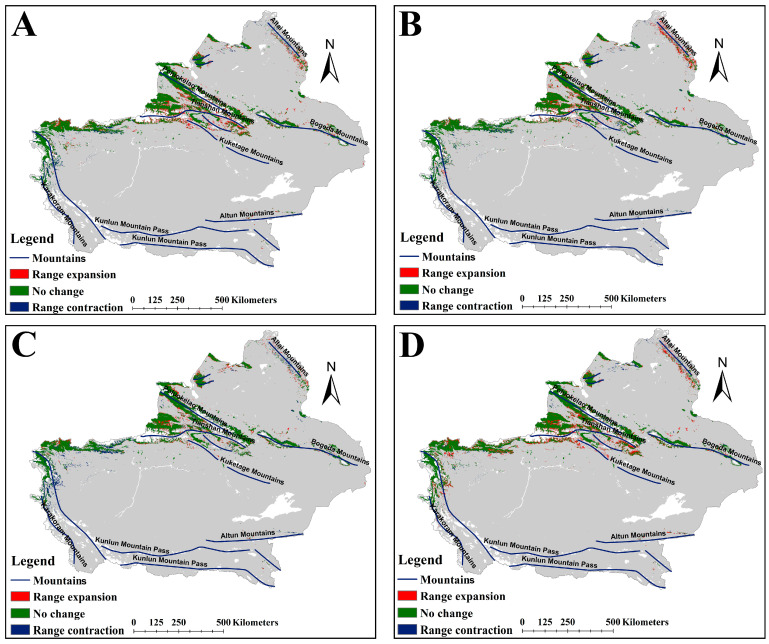
Changes in suitable habitats for *A. euchroma* under future climate scenarios. (**A**) ssp126-2050s, (**B**) ssp585-2050s, (**C**) ssp126-2090s, (**D**) ssp585-2090s.

**Figure 5:**
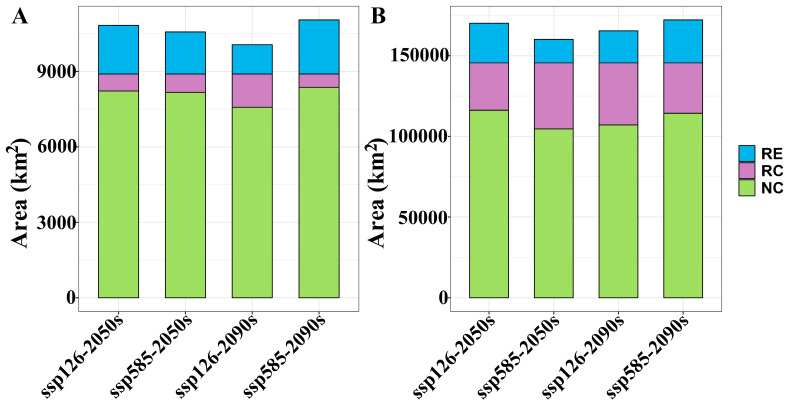
The areas of range expansion, range contraction, and no change in suitable habitats for two medicinal plants under future climate scenarios. (**A**) *A. euchroma*, (**B**) *A. guttata*. RE: range expansion, RC: range contraction, NC: no change.

The suitable habitat areas of *A. euchroma* under the four future climate scenarios, namely ssp126-2050s, ssp585-2050s, ssp126-2090s, and ssp585-2090s, were 124,780, 121,283, 107,568, and 128,839 km^2^, respectively (Table 2). The suitable habitat area under the ssp126-2090s scenario decreased, while under the other scenarios the area increased. The spatial distribution patterns of suitable habitats under future climates remained broadly consistent with the current scenario (Figure 3), though with localized expansions or contractions relative to present-day ranges (Figure 4). Habitat suitability was also similar to the current scenario (Figure S2). Under the future climate scenarios, the areas of suitable habitat for *A. euchroma* would expand by 19,399, 16,731, 11,665, and 21,560 km^2^, respectively, while the areas of contraction would be 6725, 7264, 13,216, and 5262 km^2^, respectively (Figure 5). Notably, the area of suitable habitat contraction in the ssp126-2090s scenario was greater than the area of expansion, resulting in a decrease in the area of suitable habitats in this scenario compared to the current area. Conversely, the area of suitable habitat expansion in the ssp585-2090s scenario was much greater than the area of contraction, resulting in the largest area of suitable habitats in this scenario.

The expansion or contraction of suitable habitats under future climate scenarios occurred in the same areas. The expansion of suitable habitats was mainly distributed in the northern and southern slopes of Tianshan Mountains and the western slope of Altai Mountains, while the contraction of suitable habitats was mainly distributed in the eastern and western areas of the northernmost Kunlun Mountain Pass (Figure 4). However, there were certain differences between the different scenarios. For example, the expansion of suitable habitats in the ssp585-2090s scenario was also clearly distributed in the northernmost part of the Kunlun Mountain Pass (Figure 4D).

**Figure 6:**
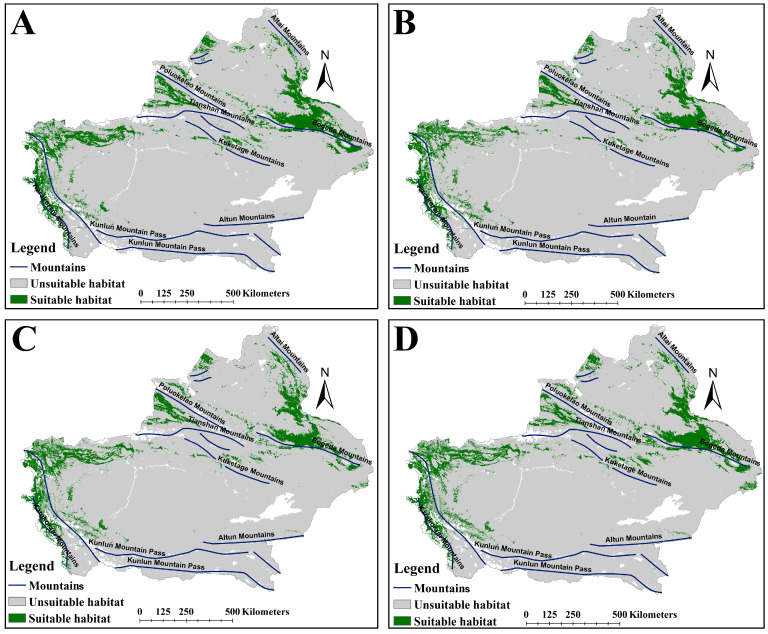
The suitable habitat distribution map of *A. guttata* under future scenarios. (**A**) ssp126-2050s, (**B**) ssp585-2050s, (**C**) ssp126-2090s, (**D**) ssp585-2090s.

**Figure 7:**
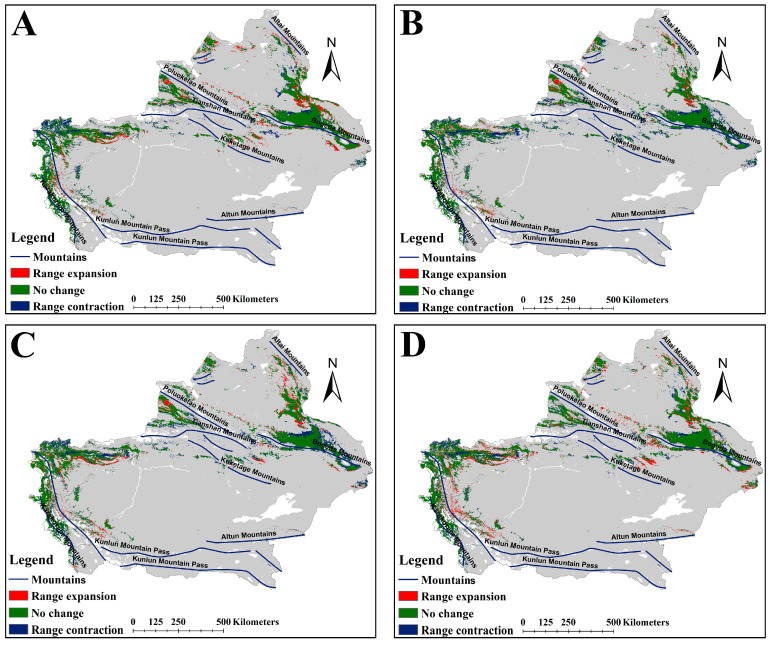
Changes in suitable habitats for *A. guttata* under future climate scenarios. (**A**) ssp126-2050s, (**B**) ssp585-2050s, (**C**) ssp126-2090s, (**D**) ssp585-2090s.

The suitable habitats areas of *A. guttata* under the future climate scenarios were 171,081, 144,670, 153,920, and 170,757 km^2^, respectively (Table 2). Compared with the current scenario, the suitable habitat areas decreased, with the ssp585-2050s scenario experiencing the most significant reduction. The future distribution pattern of suitable habitats for *A. guttata* was similar to the current pattern (Figure 6), but with some expansion or contraction (Figure 7). Notably, the ssp585-2050s scenario experienced a sharp decline in habitat suitability, whereas the other three scenarios maintained suitability levels comparable to the present (Figure S3). Quantitative analysis revealed expansion areas of 24,616, 14,534, 19,811, and 26,657 km^2^, paired with corresponding shrinkage areas of 29,356, 40,883, 38,407, and 31,228 km^2^ across the scenarios (Figure 5). The area of suitable habitat shrinkage was greater than the expansion, resulting in the area of suitable habitat for *A. guttata* in the future climate scenario being lower than the current area. The ssp585-2050s scenario exhibited the smallest expansion and most pronounced contraction, leading to the smallest suitable habitat area among all scenarios.

Like *A. euchroma*, *A. guttata* exhibited similar spatial patterns in suitable habitat expansions and contraction under future climate scenarios (Figure 7). Expanded suitable habitats were predominantly distributed across the northern part of the Bogeda Mountains, the western and southern part of the Altai Mountains, the area between the Tianshan Mountains and the Poluokelao Mountains, the northern part of the Kugetage Mountains, and the eastern part of the northern section of the Kunlun Mountain Pass. Contracted suitable habitats were concentrated in areas north of the Bogeda Mountains and south of the Altai Mountains as well as the eastern part of the northern section of the Kunlun Mountain Pass (Figure 7). There were some differences between the different scenarios. For example, for the ssp585-2050s scenario, there was almost no expansion of suitable habitats on the northern slope of the Kuketage Mountains, but there was a reduction in suitable habitats (Figure 7B). For the other scenarios, both expansion and reduction of suitable habitats occurred in this area (Figure 7A,C,D).

### 2.4. Effects of LULC on the Distribution of Suitable Habitats for Two Medicinal Plants

**Figure 8:**
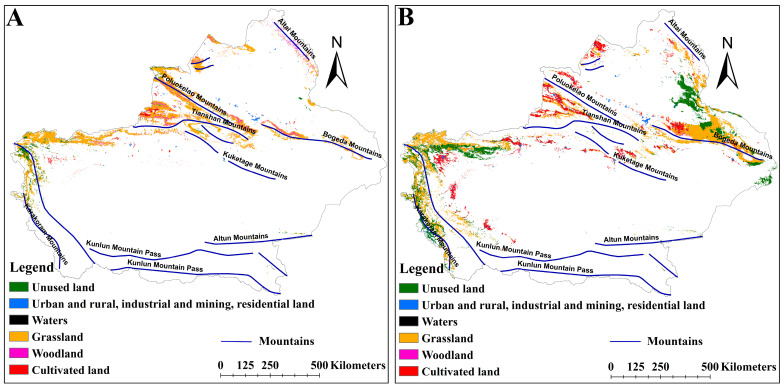
Distribution of suitable habitats for two medicinal plants in different LULCs under the current scenario. (**A**) *A. euchroma*, (**B**) *A. guttata*.

**Figure 9:**
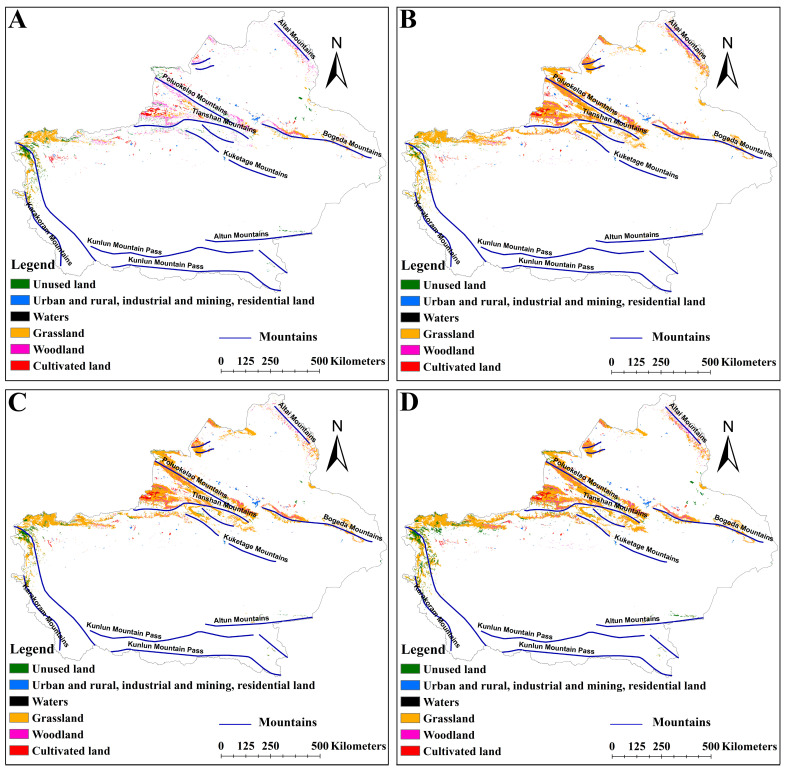
Distribution of suitable habitats for *A. euchroma* in different LULCs under future climate scenarios. (**A**) ssp126-2050s, (**B**) ssp585-2050s, (**C**) ssp126-2090s, (**D**) ssp585-2090s.

**Figure 10:**
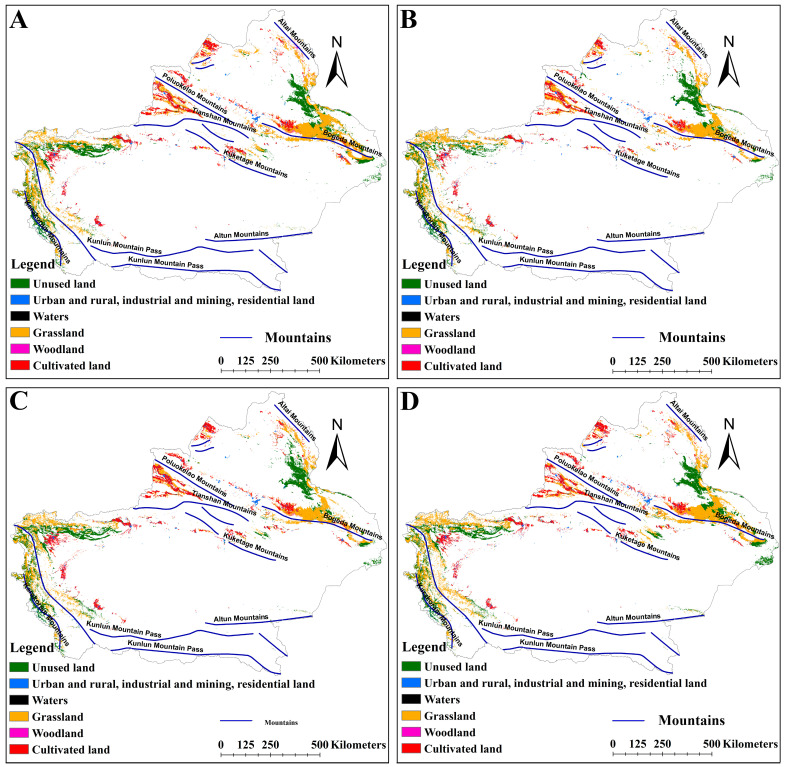
Distribution of suitable habitats for *A. guttata* in different LULCs under future climate scenarios. (**A**) ssp126-2050s, (**B**) ssp585-2050s, (**C**) ssp126-2090s, (**D**) ssp585-2090s.

**Figure 11:**
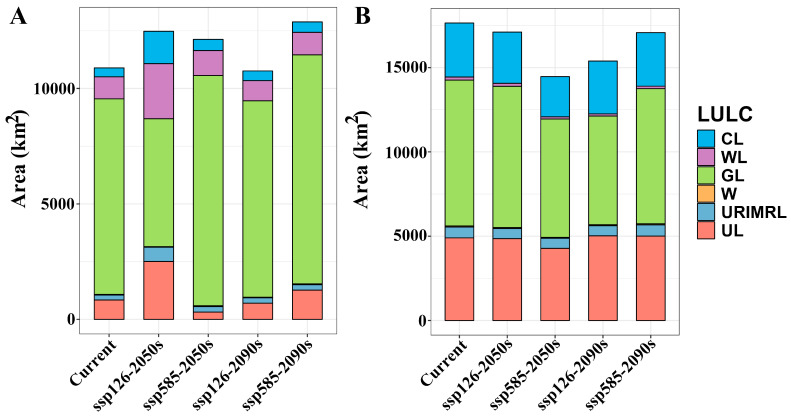
Distribution areas of suitable habitat for two medicinal plants in different LULCs under different climate scenarios. (**A**) *A. euchroma*, (**B**) *A. guttata*. LULC: land use/land cover, CL: cultivated land, WL: woodland, GL: grassland, W: waters, URIMRL: urban and rural, industrial and mining, residential land, UL: unused land.

Under different climate scenarios, the distribution of suitable habitats in different LULCs was different (Figure 8, Figure 9 and Figure 10). The suitable habitats of *A. euchroma* under the current and future climate scenarios were mainly distributed in grasslands (Figure 8A and Figure 9). The suitable habitat areas in grasslands in the current, ssp126-2050s, ssp585-2050s, ssp126-2090s, and ssp585-2090s scenarios were 84,710, 55,388, 99,711, 85,062, and 99,182 km^2^, respectively (Figure 11A). The suitable habitats in other LULCs were relatively small. Notably, the ssp126-2050s scenario exhibited a decrease in grassland-based suitable habitat area, while concurrent increases were observed in cultivated land, woodland, and unused land categories (Figure 9A and Figure 11A).

The distribution of suitable habitats for A. guttata across different LULCs exhibited slight divergence from *A. euchroma* (Figure 8B and Figure 10). The proportion of suitable habitats for *A. guttata* in different LULCs was relatively stable under different climate scenarios, but it was still mainly distributed in grasslands, followed by unused land and cultivated land, and the proportion in other LULCs was very small (Figure 8B, Figure 10 and Figure 11B). The areas in grasslands were 86,574, 83,736, 70,223, 64,483, and 80,197 km^2^; the areas in unused land were 49,002, 48,503, 42,808, 50,202, and 50,047 km^2^; and the areas in cultivated land were 32,036, 30,420, 23,872, 31,365, and 31,772 km^2^ (Figure 11B).

### 2.5. Relationship Between Secondary Metabolite Contents of Two Medicinal Plants and Habitat Suitability and Environmental Variables

**Figure 12:**
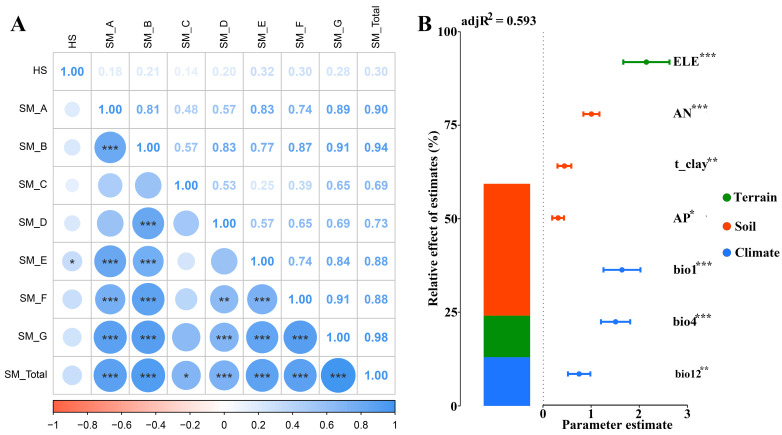
Relationship between the content of secondary metabolite and habitat suitability as well as environmental variables. (**A**) Relationship between secondary metabolite content and habitat suitability. (**B**) Relationship between secondary metabolite content and environmental variables. HS: habitat suitability, SM_A: L-shikonin, SM_B: Acetylshikonin, SM_C: β-Acetoxyisovalerylalkannin, SM_D: Deoxyalkannin, SM_E: Isobutylshikonin, SM_F: β,β’-dimethylacrylamine, SM_G: 2-methylbutylshikonin, SM_Total: total secondary metabolites. ELE: elevation, AN: available nitrogen content, t_clay: soil clay fraction, AP: available phosphorus content, bio1: annual mean temperature, bio4: temperature seasonality, bio12: annual precipitation. * *p* < 0.05; ** *p* < 0.005; *** *p* < 0.001.

The analysis of phytochemical suitability has important implications for the planning of suitable planting areas for the two *Arnebia* species. Therefore, this study analyzed the relationship between secondary metabolite content and habitat suitability (Figure 12A). The results showed that the contents of seven secondary metabolites in the roots of the two medicinal plants and the total secondary metabolites showed a certain positive correlation with habitat suitability, especially SM_E, which showed a significant positive correlation (*p* < 0.05) (Figure 12A).

In addition, the relationship between secondary metabolite content and environmental variables was further analyzed. Since the content of total secondary metabolites was significantly positively correlated with the seven secondary metabolites, the content of total secondary metabolites was used as an example for analysis. The results showed that the content of total secondary metabolites was positively correlated with elevation (*p* < 0.001), AN (*p* < 0.001), t_clay (*p* < 0.005), AP (*p* < 0.05), bio1 (*p* < 0.001), bio4 (*p* < 0.001), and bio12 (*p* < 0.01) (Figure 12B, Table S1). However, different environmental factors had different effects on the content of total secondary metabolites. Overall, soil factors (35.25%) had the greatest influence on the content of total secondary metabolites, followed by climate (13.04%), while terrain (11.05%) had the least influence (Table S2).

**Figure 13:**
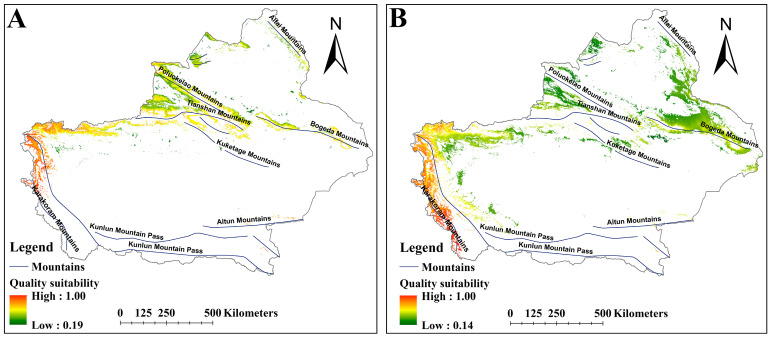
The distribution of suitable habitat for the two medicinal plants under the current climate scenario. (**A**) *A. euchroma*, (**B**) *A. guttata*.

According to the results of the model (Table S1), the phytochemical suitability distribution maps of the two species under the current and future climate scenarios were generated (Figure 13 and Figures S4 and S5). The results showed that *A. euchroma* currently has higher phytochemical suitability in the south of the Tianshan Mountains and the eastern and western areas of the northernmost part of the Kunlun Mountain Pass. Overall, suitable habitats in western Xinjiang had higher phytochemical suitability for *A. euchroma* (Figure 13A). The pattern of phytochemical suitability for *A. euchroma* under future climate scenarios was similar to the current pattern (Figure S4). The phytochemical suitability of *A. guttata* was different from that of *A. euchroma*. In the suitable habitats of *A. guttata*, the western region of Xinjiang, specifically the eastern slope of the Karakoram Mountains and the northernmost part of the Kunlun Mountain Pass, had higher phytochemical suitability (Figure 13B and Figure S5). Comparison of the distribution maps of habitat and quality suitability showed that they were relatively high in the western region of Xinjiang, including the eastern and western sides of the Kunlun Mountain Pass and the eastern slope of the Karakoram Mountains (Figure 13 and Figures S1–S5).

## 3. Discussion

### 3.1. Changes in Suitable Habitats of the Two Medicinal Plants

Future climate change will result in an increase or decrease in the area of suitable habitat for certain medicinal plants. Climate warming has expanded the suitable habitats of *Ophiocordyceps sinensis* (Berk.) G.H. Sung, J.M. Sung, and Hywel-Jones & Spatafora and improved their quality [34]. However, future climate would cause a decrease in the suitable habitats of *Nardostachys jatamansi* (D. Don) DC, especially highly suitable habitats [25]. This study examined how the suitable habitats of the two *Arnebia* species would change in the future. For *A. euchroma*, except for the ssp126-2090s scenario, future climate change would be favorable for its growth, but the suitable habitats would tend to move to higher latitudes (Figure 4). However, future climate change would reduce the suitable habitats of *A. euchroma*, which is not conducive to its growth.

Previous studies have also predicted the distribution of suitable habitats for *A. euchroma*. The results of these predictions differ due to variations in research areas, species occurrence records, environmental variables, and models used. Nevertheless, there are also certain similarities [35,36]. All studies have found that the suitable habitats for *A. euchroma* were distributed across the Tianshan and Altai Mountains. However, this study collected as many species occurrence records as possible from databases and the literature, resulting in a broader prediction of suitable habitats. There is no report on the prediction of suitable habitats for *A. guttata*. Its roots can also be used as the medicinal herb *Arnebiae Radix*, and predicting suitable habitats for *A. guttata* has implications for its utilization.

### 3.2. Environmental Factors Affecting the Distribution of Suitable Habitats and Secondary Metabolites of Two Medicinal Plants

Ecological and environmental factors not only affect the distribution of suitable habitats for medicinal plants but also influence the synthesis or accumulation of their secondary metabolites and even their medicinal value [23,28,37]. Different plant habitats are affected by different factors. *A. euchroma* mainly grows on gravel slopes, alluvial fans, grasslands and meadows, while *A. guttata* grows on Gobi, rocky slopes and lakeside gravels. The prediction results showed that both medicinal plants were affected by land use/land cover and soil available nitrogen, but *A. euchroma* was also affected by the precipitation in the driest month. The growth area of *A. guttata* is unique and has low land development potential, so it is slightly less affected by LULC than *A. euchroma*. In addition, the soil nutrient content in such habitats is low, so the soil nutrient content, such as a change of available nitrogen content, is bound to have an impact on its growth. Furthermore, the characteristics of the two *Arnebia* species themselves will also cause differences in the factors affecting their suitable habitat distribution. *A. euchroma* is a perennial herb with extremely low seed yield in the natural environment, whereas *A. guttata* is an annual herb with relatively high seed yield [38]. Therefore, *A. guttata* is more likely to spread in the natural environment. Such characteristics have caused LULC to have a greater impact on the suitable habitat distribution of *A. guttata*.

The key factors affecting the distribution of suitable habitats for the two medicinal plants in this study differ from those in other studies. One study found that the distribution of suitable habitats for *A. euchroma* was mainly affected by elevation, mean temperature in October, precipitation in June, precipitation in December, soil type, vegetation type, and isothermality [36]. Another study on *A. euchroma* showed that the mean temperature in the wettest quarter and the mean temperature in the warmest quarter mainly affected the distribution of its suitable habitats [35], which differs from the results of this study. This may be due to differences in the selection of study areas and environmental variables. In this study, factors such as land use/land cover and soil nutrients were introduced, and the suitable habitats of two medicinal plants were greatly affected by land use/land cover, resulting in less influence of topographic and bioclimatic variables. The precipitation in the driest month and the mean diurnal range of climatic factors affected the future distribution of suitable habitats. Changes to these two factors in the future may cause the expansion or contraction of suitable habitats. Mountains provide an excellent opportunity for many species to migrate to high altitudes to mitigate the effects of high temperature [39]. The suitable habitats of the two medicinal plants in this study were mainly distributed along the mountains of Xinjiang. However, the elevation was not the main factor affecting the distribution of suitable habitats. The results also showed that the suitable elevation range under future climate scenarios remained unchanged (Figure S6).

The content of secondary metabolites in *Arnebiae Radix* was affected by different environmental variables associated with suitable habitat distribution. The content of secondary metabolites was affected by terrain, soil, and climate, but soil factors had the greatest effect. All seven environmental variables predicted by the regression model played a role in promoting the accumulation of total secondary metabolites. In addition to genetic control, the synthesis of secondary metabolites in medicinal plants is also influenced by various environmental factors. The main secondary metabolites in *Arnebiae Radix* are naphthoquinone compounds. Currently, few studies have examined their relationship with environmental factors. A study of wild *A. euchroma* revealed a positive correlation between the soil available phosphorus content and the content of total hydroxynaphthoquinone pigments and β,β′-dimethylacrylalkannin in the roots of *A. euchroma* [40]. This is consistent with the results of this study and indicates that an increase in soil phosphorus content may contribute to an increase in the content of naphthoquinone compounds in the roots of *A. euchroma*. The same category of secondary metabolites in different plants may have different relationships with environmental factors. Research findings on *Rubia cordifolia* L. revealed a positive correlation between naphthoquinone compounds and soil available phosphorus, available potassium, available nitrogen, and organic matter content [41], which is consistent with the results of this study. Studies have confirmed that phosphorus plays a key role in the synthesis of secondary metabolites in medicinal plants, especially carbon-based secondary metabolites. On the one hand, higher phosphorus content in the soil can promote phosphorus absorption by plant roots and increase the carbon–nitrogen ratio of plant roots, thereby promoting the distribution of plant carbon to secondary metabolite synthesis [42]. On the other hand, phosphorus is directly involved in the synthesis of some secondary metabolite precursors and the catalytic reaction of related enzymes. Phosphorus plays an important role in the biosynthesis of terpenoids and flavonoids through the mevalonic acid (MVA) pathway and methylerythritol phosphate (MEP) pathway, and the absorption of phosphorus can increase the synthesis of pyrophosphate compounds such as acetyl-CoA, glyceraldehyde phosphate, and pyruvate [43,44]. The synthesis of shikonin compounds in the roots of A. euchroma involves the MVA pathway and MEP pathway [45], proving the importance of soil phosphorus content in the synthesis of secondary metabolites in the roots of the two *Arnebia* species. However, the mechanism behind the relationship between other climatic factors and soil factors and the content of secondary metabolites in the roots of the two *Arnebia* species requires further study.

### 3.3. Impact of LULC on the Distribution of Suitable Habitats

LULC has an important impact on the distribution of suitable habitats for species. The rapid growth of the global economy and trade has led to a drastic increase in human activities, resulting in significant changes to LULC. This transformation of land from suitable to unsuitable habitats hinders the survival of species [46,47]. Analyzing the distribution of suitable habitats in different LULCs can help to protect and utilize species. In this study, the distribution patterns of suitable habitats for the two medicinal plants differed among the five LULCs. Although the suitable habitats for the two medicinal plants tended to be found in grasslands, suitable habitats for *A. guttata* were also relatively prevalent in cultivated land and unused land. The distribution of suitable habitats for *A. guttata* in different LULCs under the five climate scenarios was relatively stable, facilitating the protection and utilization of *A. guttata*. The distribution of suitable habitats for *A. euchroma* varies greatly among different LULCs. In the ssp126-2050s scenario, the distribution of suitable habitats decreased in cultivated land while increasing in cultivated land, forest land, and unused land. However, grassland still dominated in the remaining scenarios. Due to the government’s emphasis on the protection of grassland resources, such a distribution pattern suggests certain limitations in the utilization of *A. euchroma*.

### 3.4. Suggestions on the Protection and Utilization of Two Medicinal Plants Combined with Habitat Suitability and Quality 

Habitat suitability is defined as the extent to which the habitat range predicted by SDMs is conducive to plant growth. Phytochemical suitability, on the other hand, is defined as the level of secondary metabolites in medicinal plants within the study area. Predicting the distribution of suitable habitats for medicinal plants enables their protection and utilization. However, suitable habitats do not necessarily produce high-quality Chinese medicinal materials [26]. Secondary metabolites are essential for medicinal plants to be effective. Therefore, establishing the relationship between the content of secondary metabolites and environmental factors, and generating a phytochemical suitability distribution map, can more accurately predict suitable planting areas [8,48]. For example, comparing the suitable habitat distribution map with the spatial distribution map of the phytochemical suitability of *Codonopsis pilosula* (Franch.) Nannf. revealed consistency, indicating that *C. pilosula* could be cultivated in highly suitable habitats [5]. This study showed that the content of secondary metabolites was positively correlated with habitat suitability, and the spatial distribution map of phytochemical suitability was also consistent with the suitable habitat distribution map. Overall, the habitat and phytochemical suitability of the two *Arnebia* species in western Xinjiang are favorable and their distribution patterns remain unchanged under future climate scenarios.

Since most of the current medicinal plants rely on field excavation, many are at risk of extinction. This makes the protection of medicinal plants, especially those with a small distribution range, very important. *A. euchroma* and *A. guttata* are mainly found in Xinjiang in China, and their distribution range is relatively small. Furthermore, the two *Arnebia* species on the market mainly originate from two wild resources, which has led to *A. euchroma* being designated a nationally protected species. Therefore, it is crucial to implement measures to safeguard its survival. Medicinal plants can be protected by various measures, such as the establishment of protected areas to protect wild populations [49]. For the two *Arnebia* species, areas with high habitat and phytochemical suitability, such as grassland in the western region of Xinjiang, should be given priority protection. In addition, the two *Arnebia* species in these areas should be actively collected to preserve DNA resources [49,50], and sequencing should be carried out to study the synthesis pathway of secondary metabolites in order to improve their quality through genetic engineering. However, conservation does not mean that these resources cannot continue to be used. Therefore, it is possible to choose an appropriate area for the standardized cultivation of the two species. Where policy allows, farmland or unused land in areas with higher habitat and phytochemical suitability would be preferable. It is also an effective protection measure to reduce the excavation of wild resources by standardizing the planting of the two *Arnebia* species.

However, the standardized cultivation of medicinal plants also requires the implementation of appropriate measures, such as reasonable fertilization and irrigation. The shikonin compounds in the roots of the two *Arnebia* species selected in this study belong to carbon-based secondary metabolites. The results of this study showed that the content of total secondary metabolites was correlated with climate, topography, and soil factors, especially soil factors. Therefore, for medicinal plants growing in high-altitude areas and dominated by carbon-based secondary metabolites, regulating soil nutrient content, including available nitrogen and phosphorus, can improve quality while ensuring yield. This result has important implications for the future standardized planting of *Arnebia* species and similar plants.

## 4. Materials and Methods

### 4.1. Study Area

Xinjiang is located in the heart of Eurasia, in the northwest of China, with a vast area of 166 million hm^2^. It is the largest provincial administrative region in China in terms of land area. Xinjiang belongs to the typical temperate continental arid climate, with an average annual temperature of 4–8 °C in northern Xinjiang and 10–13 °C in southern Xinjiang. The annual sunshine hours are 2550–3500 h, and the annual precipitation is 100–200 mm in northern Xinjiang and 20–100 mm in southern Xinjiang. The frost-free period is 180–220 d. The annual evaporation is 1500–2300 mm in northern Xinjiang and 2100–3400 mm in southern Xinjiang [51]. Xinjiang has a complex and diverse terrain, with glaciers, basins, valleys, rivers, oases, grasslands, and other landforms. These provide a variety of ecological environments for the survival and development of medicinal plant species and also make the distribution of medicinal plants in Xinjiang more extensive [52].

### 4.2. Materials

#### 4.2.1. Species Occurrence Records

**Figure 14:**
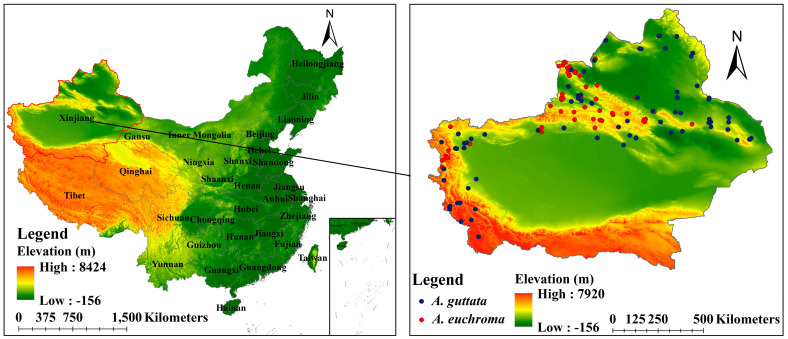
Occurrence records of two *Arnebia* species.

The occurrence records of the two *Arnebiae* species were obtained through multiple channels, including the Global Biodiversity Information Facility (GBIF; https://www.gbif.org/, accessed on 1 May 2024), the Chinese Virtual Herbarium (CVH; https://www.cvh.ac.cn, accessed on 7 May 2024), and the literature [40,53,54,55]. The spThin package [56] in R 4.2.2 was used to reduce the occurrence records of the two *Arnebia* species to only one record per grid (1 km × 1 km) to minimize sampling bias. Finally, there were 61 occurrences of *A. euchroma* and 90 occurrences of *A. guttata* (Table S3, Figure 14).

#### 4.2.2. Environmental Variables

To predict the suitable habitats for both medicinal plants, various environmental variables, including bioclimatic variables, geographical factors, soil factors, and land use/land cover (LULC) were selected. A total of 19 bioclimatic variables and elevation data were obtained from the WorldClim data website (https://www.worldclim.org/, accessed on 7 May 2024) [57], and the two Shared Socio-economic Pathways ssp126 and ssp585 of future climate scenarios in two time periods of 2041–2060 (2050s) and 2081–2100 (2090s) were selected. The ssp126 scenario represents the lowest CO_2_ emissions in the future, while the ssp585 scenario represents the highest CO_2_ emissions in the future. The BCC-CSM2-MR (Beijing Climate Center Climate System Model) from CMIP6 (Coupled Model Intercomparison Project Phase 6) was selected as the global climate model (GCM). The slope and aspect in the terrain data were converted from the elevation data by ArcGIS 10.7. The nutrient indicators in soil factors including available phosphorus (AP), available potassium (AK), available nitrogen (AN), and soil organic matter content (SOM) were obtained from the National Tibetan Plateau/Third Pole Environment Data Center (https://data.tpdc.ac.cn/, accessed on 7 May 2024) [58], while soil pH, soil sand content (t_sand), and soil clay composition (t_clay) were obtained from the soil map-based Harmonized World Soil Database (v1.2) (https://data.tpdc.ac.cn/, accessed on 7 May 2024) [59]. LULC data were obtained from the Resource and Environmental Science and Data Platform (https://www.resdc.cn/, accessed on 7 May 2024) [60]. All environmental variables have a resolution of 30 s.

The 19 bioclimatic variables were analyzed for collinearity problems, and their correlation coefficients were calculated using SDMtoolbox [61] in AricGIS 10.7. Two variables with correlation coefficients |r| greater than 0.8 and with less ecological significance were discarded [24]. Finally, seven bioclimatic variables closely related to plant growth, including annual mean temperature (bio1), mean diurnal range (bio2), isothermality (bio3), temperature seasonality (bio4), annual precipitation (bio12), precipitation of driest month (bio14), and precipitation seasonality (bio15), were retained. In addition, the remaining 12 bioclimatic factors were excluded because of their low ecological significance and significant correlation with the above seven bioclimatic factors. Therefore, the final 18 environmental variables were used to predict the suitable habitats of the two medicinal plants (Table S4).

### 4.3. Methods

#### 4.3.1. Model Construction

In this study, MaxEnt 3.4.4 was used to predict suitable habitats for the two medicinal plants. Model default parameters were retained, including feature type combinations and regularization multiplier settings. In addition, 25% of the occurrence records of the two medicinal plants were used as test sets and 75% as training sets. The number of repetitions was ten. The contribution rate of each environmental variable was calculated using the Jackknife test in MaxEnt. Model performance was evaluated using AUC (area under the receiver operating characteristic curve) and TSS (true skill statistic). AUC values range from 0 to 1, and the accuracy and reliability of the model increase with increasing AUC values [62]. TSS is a threshold-related measure of sensitivity and specificity, with values ranging between −1 and 1; a TSS value below 0.4 indicates poor model performance [63].

Following model execution in MaxEnt, the potential habitat distribution maps for the two medicinal plant species were generated. The potential habitats were divided into suitable habitats and unsuitable habitats using the MSS threshold (the value that maximized the sum of sensitivity and specificity). Previous studies have shown that this threshold can divide the potential habitats more reasonably [64]. The suitable habitat maps and habitat change maps for both species were generated using ArcGIS 10.7. The stacked bar chart of habitat change area and the wind rose diagram of environmental variable contribution rate were generated using the ggplot2 package via R 4.2.2.

#### 4.3.2. Analysis of Suitable Habitat Distribution Under Different LULCs

To analyze the distribution of suitable habitats under different LULCs, the LULC was divided into five types, namely, cultivated land, woodland, grassland, waters, urban and rural areas, industrial and mining, residential land, and unused land. The suitable habitat distribution maps for the two medicinal plants were overlaid with LULC maps to quantify habitat occurrence in each class. ArcGIS 10.7 was used to map habitat-LULC overlays, and the ggplot2 package was used to generate a stacked bar chart of the area of suitable habitats in different LULCs via R 4.2.2.

#### 4.3.3. Analysis of the Relationship Between Secondary Metabolite Content and Habitat Suitability

Analyzing the relationship between secondary metabolite content and habitat is critical for optimizing suitable planting areas for the two medicinal plant species. Therefore, the secondary metabolite content of the two medicinal plants was collected through the literature. In order to maintain the consistency of the study, the content of seven secondary metabolites in the roots of the two medicinal plants was collected through screening [53]. There were 18 data sets for each of the two species, including the geographical coordinates of the sampling sites (Table S5). Then, habitat suitability and environmental variables of the sampling sites were extracted using ArcGIS 10.7 (Tables S5–S7). A correlation heat map of habitat suitability and secondary metabolite content was generated in R 4.2.2 using the corrplot package.

The growth and quality of medicinal plants are influenced by multiple environmental factors [65,66]. The relationship between the content of secondary metabolites and environmental factors was analyzed. In this study, the MuMIn R package was employed to perform full subset multiple regression to analyze the correlation between the total content of secondary metabolite in the roots of two *Arnebia* species and environmental variables. Full subset regression offers the advantage of evaluating all possible model combinations, making it generally more robust than stepwise regression approaches. The adjusted R^2^ (adjR^2^) was used to assess model fit, while *p*-values were employed to determine the statistical significance of relationships between environmental factors and active ingredient content [28,67,68].

## 5. Conclusions

In this study, the suitable habitats of two *Arnebia* species were predicted based on the MaxEnt model, and the relationships between the secondary metabolite contents and habitat suitability and environmental factors were analyzed. Meanwhile, a spatial distribution map of phytochemical suitability was generated. The results showed that the suitable habitats of the two medicinal plants were mainly distributed along the main mountains in Xinjiang under the current and future climate scenarios. The suitable habitats of *A. guttata* were found to be more extensive than those of *A. euchroma*. The suitable habitats of *A. euchroma* tended to expand in the future, while those of *A. guttata* tended to shrink. The suitable habitats of the two species were mainly distributed in grasslands, but a large proportion of the suitable habitats of *A. guttata* were distributed in cultivated land and unused land. Therefore, the protection and utilization of *A. guttata* would be beneficial in the future, while the protection and utilization of *A. euchroma* would be subject to certain restrictions. The distribution of suitable habitats and secondary metabolites were affected by different environmental variables. However, the content of secondary metabolites was positively correlated with habitat suitability, and the spatial distribution of quality suitability was consistent with habitat suitability. The eastern and western areas of the northern Kunlun Mountain Pass were the key focus areas for the two medicinal plants, with a high overlap in habitat suitability and quality suitability. This study will provide a scientific basis for the protection and utilization of the *Arnebia* resources in Xinjiang.

## Figures and Tables

**Table 1:** MaxEnt model performance for *A. euchroma* and *A. guttata*.

Indicator	*A. euchroma*	*A. guttata*
AUC	0.977	0.952
TSS	0.829	0.725

**Table 2:** The suitable and unsuitable habitat areas of two medicinal plants under different climate scenarios.

Scenario	*A. euchroma*		*A. guttata*	
	Unsuitable Habitat (km^2^)	Suitable Habitat (km^2^)	Unsuitable Habitat (km^2^)	Suitable Habitat (km^2^)
Current	1,522,816	108,914	1,455,285	176,445
ssp126-2050s	1,506,950	124,780	1,460,649	171,081
ssp585-2050s	1,510,447	121,283	1,487,060	144,670
ssp126-2090s	1,524,163	107,568	1,477,810	153,920
ssp585-2090s	1,502,891	128,839	1,460,973	170,757

## Data Availability

The original contributions presented in this study are included in the article/Supplementary Materials. Further inquiries can be directed to the corresponding author.

## Supplementary Materials

The following supporting information can be downloaded at: https://www.mdpi.com/article/10.3390/plants14111669/s1, Figure S1: Habitat suitability distribution map of two medicinal plants under the current scenario; Figure S2: Habitat suitability distribution map of *A. euchroma* under future climate scenarios; Figure S3: Habitat suitability distribution map of *A. guttata* under future climate scenarios; Figure S4: Quality suitability distribution map of *A. euchroma* under future climate scenarios; Figure S5: Quality suitability distribution map of *A. guttata* under future climate scenarios; Table S1: Regression results between total secondary metabolite contents of two medicinal plants and environmental variables; Table S2: Explanation of topographic, soil and climate factors affecting the total secondary metabolite content of two medicinal plants; Table S3: Coordinate points for suitable habitat prediction of two *Arnebia* species; Table S4: Potential environmental variables used to predict the distribution of two medicinal plants; Table S5: Habitat suitability of sampling sites and secondary metabolite contents in roots of two medicinal plants; Table S6: Terrain and climate data of sampling points; Table S7: Soil data at sampling points.
