# Supplementary material for: Suitable Planting Area Prediction for Two Arnebia Species: An Analysis Based on Habitat and Phytochemical Suitability
Source: Plants (Basel). 2025 May 30;14(11):1669. doi: 10.3390/plants14111669 (PMC12157611; doi:10.3390/plants14111669)
Supplement: Supplementary file 1 [file plants-14-01669-s001.zip › plants-3628409-supplementary.pdf]

Table S1. Regression results between total secondary metabolite contents of two medicinal plants and environmental variables.

Envrionmental variable	Estimate	Std. Error	t value	*p*
AN	1.01	0.17	6.04	<0.001
AP	0.31	0.12	2.57	0.016
bio1	1.64	0.38	4.27	<0.001
bio12	0.75	0.23	3.23	0.003
bio4	1.51	0.30	4.97	<0.001
ELE	2.15	0.48	4.48	<0.001
t_clay	0.45	0.14	3.10	0.004

Note: AN: Available nitrogen content; AP: Available phosphorus content; bio1: Annual mean temperature; bio12: Annual precipitation; bio4: Temperature seasonality; ELE: Elevation; t_clay: Soil clay fraction.

Table S2. Explanation of topographic, soil and climate factors affecting the total secondary metabolite content of two medicinal plants.

Factor	Explanation (%)
Climate	13.04
Terrain	11.05
Soil	35.25

Table S3. Coordinate points for suitable habitat prediction of two *Arnebia* species.

Species	Longitude	Latitude	Source	ID
Arnebia euchroma	74.92	38.15	GBIF	615269332
Arnebia euchroma	75.02	38.43	GBIF	615269326
Arnebia euchroma	75.05	38.73	GBIF	615269322
Arnebia euchroma	81.0805	44.518142	GBIF	4527971915
Arnebia euchroma	75.95	39.15	GBIF	4043718601
Arnebia euchroma	84.27	41.78	GBIF	4043695680
Arnebia euchroma	81.12	43.15	GBIF	2417624603
Arnebia euchroma	75.22	37.77	GBIF	2417423811
Arnebia euchroma	87.209708	43.3254	CVH	1437962
Arnebia euchroma	87.213333	43.195556	CVH	0042826
Arnebia euchroma	83.9271	42.2551	[1]	
Arnebia euchroma	84.9121	43.1452	[1]	
Arnebia euchroma	84.8362	43.215	[1]	
Arnebia euchroma	85.8297	43.3758	[1]	
Arnebia euchroma	80.519	42.6771	[1]	
Arnebia euchroma	83.6603	42.6685	[1]	
Arnebia euchroma	80.9657	44.6102	[1]	
Arnebia euchroma	81.8888	44.6102	[1]	
Arnebia euchroma	82.5317	44.1605	[1]	
Arnebia euchroma	80.4659	44.8808	[1]	
Arnebia euchroma	82.6164	45.6878	[1]	
Arnebia euchroma	87.1698	43.2661	[1]	
Arnebia euchroma	75.0454	38.3635	[1]	
Arnebia euchroma	82.8534	43.1365	[1]	
Arnebia euchroma	82.2199	42.7349	[1]	
Arnebia euchroma	81.5213	44.7368	[1]	
Arnebia euchroma	81.4264	44.7466	[1]	
Arnebia euchroma	81.5188	44.7092	[1]	
Arnebia euchroma	80.8417	44.8067	[1]	
Arnebia euchroma	80.5249	45.0596	[1]	
Arnebia euchroma	80.589	45.0467	[1]	
Arnebia euchroma	80.5069	45.0507	[1]	
Arnebia euchroma	81.1372	44.7955	[1]	
Arnebia euchroma	80.9918	44.9151	[1]	
Arnebia euchroma	81.4238	44.7154	[1]	
Arnebia euchroma	75.2039	38.38	[1]	
Arnebia euchroma	84.2234	42.7078	[2]	
Arnebia euchroma	81.0764	43.2275	[2]	
Arnebia euchroma	87.1187	43.1896	[2]	
Arnebia euchroma	75.4879	37.2314	[2]	
Arnebia euchroma	86.219731	44.303753	[3]	
Arnebia euchroma	90.519386	43.55595	[3]	

Arnebia euchroma	82.415381	42.369739	[3]	
Arnebia euchroma	83.936367	43.169789	[3]	
Arnebia euchroma	75.081167	37.832975	[3]	
Arnebia euchroma	74.579111	39.910147	[3]	
Arnebia euchroma	80.538342	45.038378	[3]	
Arnebia euchroma	84.120364	42.700139	[3]	
Arnebia euchroma	81.164953	44.458997	[3]	
Arnebia euchroma	81.327506	42.565667	[3]	
Arnebia euchroma	82.388639	43.595083	[3]	
Arnebia euchroma	81.068367	43.443064	[3]	
Arnebia euchroma	83.468386	43.698722	[3]	
Arnebia euchroma	87.124631	43.288636	[3]	
Arnebia euchroma	83.262419	44.395167	[3]	
Arnebia euchroma	80.492006	41.259244	[3]	
Arnebia euchroma	76.209189	39.462083	[3]	
Arnebia euchroma	80.404033	41.490658	[3]	
Arnebia euchroma	81.216667	43.25	[3]	
Arnebia euchroma	87.7	43.8	[3]	
Arnebia euchroma	84.266667	42.7	[3]	
Arnebia guttata	78	35.83	GBIF	615271158
Arnebia guttata	77.13	37.3	GBIF	615270745
Arnebia guttata	78.77	35.15	GBIF	615270711
Arnebia guttata	76.97	36.45	GBIF	615270571
Arnebia guttata	76.43	36.07	GBIF	615270555
Arnebia guttata	75.42	38.97	GBIF	615270525
Arnebia guttata	78	36.43	GBIF	615270406
Arnebia guttata	77.42	35.58	GBIF	615270403
Arnebia guttata	76.95	36.45	GBIF	615270380
Arnebia guttata	76.45	36.05	GBIF	615270375
Arnebia guttata	76.8	36.4	GBIF	615270366
Arnebia guttata	76.45	36.12	GBIF	615270315
Arnebia guttata	74.43	39	GBIF	615269972
Arnebia guttata	75.52	38.98	GBIF	615269967
Arnebia guttata	75.23	37.78	GBIF	615269934
Arnebia guttata	88.13	47.85	GBIF	4045540446
Arnebia guttata	89.58	44.02	GBIF	4045460618
Arnebia guttata	81.83	43.22	GBIF	4045404659
Arnebia guttata	81.32	43.92	GBIF	4045321470
Arnebia guttata	83.25	43.43	GBIF	4045274522
Arnebia guttata	75.85	39.38	GBIF	4045101301
Arnebia guttata	81.87	41.8	GBIF	4045091998
Arnebia guttata	76.17	39.72	GBIF	4045076058
Arnebia guttata	75.95	39.15	GBIF	4045076057
Arnebia guttata	86.85	47.7	GBIF	4045058895

Arnebia guttata	94.7	43.25	GBIF	4044976091
Arnebia guttata	93.03	44.21	GBIF	4044833228
Arnebia guttata	88.3	43.35	GBIF	4044833227
Arnebia guttata	86.15	41.77	GBIF	4044668726
Arnebia guttata	85.72	46.8	GBIF	4044651231
Arnebia guttata	89.52	47	GBIF	4044484556
Arnebia guttata	82.23	43.48	GBIF	4044472124
Arnebia guttata	87.5	47.12	GBIF	4043977105
Arnebia guttata	89.18	44	GBIF	4043722704
Arnebia guttata	82.07	44.9	GBIF	4043692359
Arnebia guttata	82.98	46.75	GBIF	4043692357
Arnebia guttata	75.25	39.72	GBIF	4043683175
Arnebia guttata	84.27	41.78	GBIF	4043683174
Arnebia guttata	80.23	41.28	GBIF	4043679150
Arnebia guttata	86.87	42.27	GBIF	4043667320
Arnebia guttata	75.22	37.77	GBIF	4043662907
Arnebia guttata	86.4	42.32	GBIF	4043362202
Arnebia guttata	89.5811	47.1758	GBIF	4038686321
Arnebia guttata	86.31	44.31	GBIF	2417739118
Arnebia guttata	83.26	43.42	GBIF	2417618861
Arnebia guttata	81.87	41.79	GBIF	2417603473
Arnebia guttata	75.99	39.46	GBIF	2417407340
Arnebia guttata	86.86	42.26	GBIF	2417400121
Arnebia guttata	89.18	42.94	GBIF	2417397990
Arnebia guttata	77.41	37.88	GBIF	2417389791
Arnebia guttata	75.25	39.71	GBIF	2417292721
Arnebia guttata	91.876083	43.601389	[4]	
Arnebia guttata	91.798528	43.836111	[4]	
Arnebia guttata	91.6635	43.976778	[4]	
Arnebia guttata	93.062944	43.976778	[4]	
Arnebia guttata	93.090903	43.599306	[4]	
Arnebia guttata	94.745322	43.256361	[4]	
Arnebia guttata	76.910586	40.178342	[3]	
Arnebia guttata	89.017231	45.046114	[3]	
Arnebia guttata	91.6635	43.790444	[3]	
Arnebia guttata	82.167506	43.611397	[3]	
Arnebia guttata	75.482861	37.229083	[3]	
Arnebia guttata	91.381361	44.966808	[3]	
Arnebia guttata	86.006544	43.017192	[3]	
Arnebia guttata	85.586392	46.799742	[3]	
Arnebia guttata	89.943625	43.100558	[3]	
Arnebia guttata	82.577781	45.583336	[3]	
Arnebia guttata	91.389586	45.055569	[3]	
Arnebia guttata	90.373619	45.551953	[3]	

Arnebia guttata	81.138344	44.775575	[3]	
Arnebia guttata	82.495839	43.404728	[3]	
Arnebia guttata	82.346128	43.687508	[3]	
Arnebia guttata	93.848069	43.128508	[3]	
Arnebia guttata	94.81425	43.323697	[3]	
Arnebia guttata	89.122072	44.75485	[3]	
Arnebia guttata	86.781475	47.669453	[3]	
Arnebia guttata	74.3956333	39.9126667	CVH	TARU03290
Arnebia guttata	78.9479833	41.08695	CVH	TARU03561
Arnebia guttata	75.6636667	39.3222667	CVH	TARU00167
Arnebia guttata	74.3052667	39.8050167	CVH	TARU01214
Arnebia guttata	89.6494444	46.97	CVH	0042831
Arnebia guttata	90.2447222	46.4563889	CVH	0042834
Arnebia guttata	90.1791667	46.4919444	CVH	0042809
Arnebia guttata	89.4991667	47.0297222	CVH	0042841
Arnebia guttata	89.4975	47.0525	CVH	0042846
Arnebia guttata	85.7283333	46.7786111	CVH	0042854
Arnebia guttata	85.7025	46.7502778	CVH	0042856
Arnebia guttata	78.65975	41.2126	CVH	TARU01216
Arnebia guttata	85.6955556	46.7327778	CVH	0042808
Arnebia guttata	74.3956333	39.9026667	CVH	TARU02485

Note: GBIF: Global Biodiversity Information Facility, CVH: Chinese Virtual Herbarium.

References:

[1] Ye, Y. Effects of ecological factors on biological characteristics and quality of *Arnebia euchroma*. Master, Xinjiang Agricultural University, 2022.

[2] Zhang, J.; Qiu, Y.; Zhao, Y.; Ye, Y.; Wang, G.; Zhu, J.; Li, X.; Fan, C., Correlation between rhizosphere environment and content of medicinal components of *Arnebia euchroma*. China J. Chin. Mater. Med. 2023, 48, 6030-6038.

[3] Xu, H.; Li, P.; Ren, G.; Wang, Y.; Jiang, D.; Liu, C., Authentication of three source spices of *Arnebiae Radix* using DNA barcoding and HPLC. Front. Pharmacol. 2021, 12, 677014.

[4] Wen, E.; Liu, W.; Song, H.; Xu, H.; Tian, S., Resource Investigation of *Arnebia guttata* Bge.in Hami District of Xinjiang. Mod. Chin. Med. 2016, 18, (11), 1479-1483.

Table S4. Potential environmental variables used to predict the distribution of two medicinal plants.

Environmental variables	Abbreviation	Unit
**Annual mean temperature**	bio1	℃
**Mean diurnal range (mean of monthly (maxtemp–mintemp)**	bio2	℃
**Isothermality (bio2/bio7 × 100)**	bio3	-
**Temperature seasonality (standard deviation × 100)**	bio4	-
Max temperature of warmest month	bio5	℃
Min temperature of coldest month	bio6	℃
Temperature annual range (bio5-bio6)	bio7	℃
Mean temperature of wettest quarter	bio8	℃
Mean temperature of driest quarter	bio9	℃
Mean temperature of warmest quarter	bio10	℃
Mean temperature of coldest quarter	bio11	℃
**Annual precipitation**	bio12	mm
Precipitation of wettest month	bio13	mm
**Precipitation of driest month**	bio14	mm
**Precipitation seasonality (coefficient of variation)**	bio15	mm
Precipitation of wettest quarter	bio16	mm
Precipitation of driest quarter	bio17	mm
Precipitation of warmest quarter	bio18	mm
Precipitation of coldest quarter	bio19	mm
**Elevation**	ELE	m
**Aspect**	ASP	-
**Slope**	SLP	°
**Available phosphorus content**	AP	mg/g
**Available potassium content**	AK	mg/g
**Available nitrogen content**	AN	mg/g
**Soil organic matter content**	SOM	mg/g
**Soil pH**	t_ph	-
**Sand percentage**	t_sand	%
**Soil clay fraction**	t_clay	%
**Land use/land cover**	LULC	-

Note: The bolded environmental variables were eventually used for the prediction of the distribution of suitable habitat for two medicinal plants.

Table S5. Habitat suitability of sampling sites and secondary metabolite contents in roots of two medicinal plants.

Sepcies	Long	Lat	HS	SM_A	SM_B	SM_C	SM_D	SM_E	SM_F	SM_G	SM_Total
AE	86.22	44.30	0.31	1.45	19.85	4.47	0.36	4.89	6.68	20.83	58.52
AE	90.52	43.56	0.40	0.21	2.76	1.88	-	1.40	3.00	7.11	16.36
AE	82.42	42.37	0.29	0.25	2.70	1.28	0.23	1.20	2.98	6.57	15.21
AE	83.94	43.17	0.06	0.68	22.03	3.64	0.67	5.97	11.22	31.55	75.76
AE	75.08	37.83	0.49	0.19	1.38	8.36	-	3.19	0.60	4.12	17.83
AE	74.58	39.91	0.28	0.14	1.10	4.30	-	1.31	0.87	3.73	11.45
AE	80.54	45.04	0.85	1.07	9.43	3.67	0.37	8.55	7.99	17.19	48.27
AE	84.12	42.70	0.41	0.54	18.86	3.08	0.63	4.37	11.58	24.00	63.05
AE	81.16	44.46	0.58	0.68	12.61	5.38	0.36	7.14	4.08	16.51	46.75
AE	81.33	42.57	0.40	0.35	9.81	1.28	0.31	5.11	6.54	13.09	36.47
AE	82.39	43.60	0.09	0.80	20.04	4.21	0.64	4.20	14.25	26.26	70.39
AE	81.07	43.44	0.56	1.00	24.30	4.51	0.62	6.13	15.59	32.89	85.04
AE	83.47	43.70	0.33	0.84	25.19	3.78	0.90	5.56	12.77	28.64	77.68
AE	87.12	43.29	0.55	0.23	4.38	1.95	-	1.69	4.59	7.86	20.70
AE	83.26	44.40	0.18	0.75	12.22	4.74	0.69	6.75	5.15	15.45	45.74
AE	80.49	41.26	0.63	0.36	9.05	1.71	-	2.32	4.22	11.05	28.71
AE	76.21	39.46	0.36	0.67	9.07	2.69	0.30	2.88	5.46	14.30	35.37
AE	80.40	41.49	0.08	0.64	4.48	1.80	-	2.90	2.37	6.54	18.73
AG	76.91	40.18	0.47	0.26	6.25	6.94	0.59	0.87	1.21	4.16	20.28
AG	89.02	45.05	0.26	-	0.46	0.75	-	-	0.67	1.12	3.01
AG	91.66	43.79	0.30	-	1.10	0.87	0.26	-	0.65	1.24	4.12
AG	82.17	43.61	0.27	0.10	1.56	3.01	0.19	0.38	0.73	2.39	8.37
AG	75.48	37.23	0.33	0.57	8.13	9.79	0.58	1.99	1.33	11.13	33.51
AG	91.38	44.97	0.23	0.12	2.17	1.29	0.29	-	1.55	1.91	7.34

AG	86.01	43.02	0.12	0.11	1.56	3.16	0.21	0.55	0.95	4.05	10.59
AG	85.59	46.80	0.12	0.11	1.58	1.27	0.18	0.37	1.09	2.14	6.75
AG	89.94	43.10	0.09	0.94	2.93	14.05	0.21	1.80	1.33	14.78	36.04
AG	82.58	45.58	0.17	-	1.27	1.23	-	-	0.44	1.01	3.95
AG	91.39	45.06	0.14	0.15	3.33	0.81	0.23	-	0.55	1.08	6.15
AG	90.37	45.55	0.24	0.14	1.54	1.56	0.26	-	1.31	2.13	6.94
AG	81.14	44.78	0.14	-	0.61	1.19	-	-	0.42	0.72	2.95
AG	82.50	43.40	0.26	0.13	3.95	3.51	0.49	0.62	1.33	5.67	15.70
AG	82.35	43.69	0.18	0.17	3.57	4.41	0.26	0.56	1.48	4.63	15.08
AG	93.85	43.13	0.20	-	0.69	1.02	-	-	0.84	1.50	4.05
AG	94.81	43.32	0.32	-	0.96	1.02	0.23	-	0.82	1.44	4.47
AG	89.12	44.75	0.12	0.49	10.20	8.89	0.82	1.71	1.19	9.40	32.71

Note: AE: *A. euchroma*; AG: *A. guttata*. Long: Longitude; Lat: Latitude; HS: Habitat suitability; SM_A: L-shikonin; SM_B: Acetylshikonin; SM_C: β-Acetoxyisovalerylalkannin; SM_D: Deoxyalkannin; SM_E: Isobutylshikonin; SM_F: β,β'-dimethylacrylamine; SM_G: 2-methylbutylshikonin; SM_Total: Total secondary metabolites. "-": Not Detected.

Table S6. Terrain and climate data of sampling points.

Species	Long	Lat	ELE	ASP	SLP	bio1	bio2	bio3	bio4	bio12	bio14	bio15
AE	86.22	44.30	462	18.43	0.56	8.52	11.01	21.67	1491	152	5	44.49
AE	90.52	43.56	2464	288.33	2.71	0.24	11.65	30.10	1021	200	3	76.18
AE	82.42	42.37	2871	53.32	8.19	-1.81	11.98	29.74	1019	293	3	88.35
AE	83.94	43.17	2974	342.50	9.79	-5.59	11.08	26.77	1110	346	3	85.81
AE	75.08	37.83	4164	126.15	8.26	-3.55	12.78	32.34	978	84	1	64.62
AE	74.58	39.91	3363	146.62	9.59	-0.57	13.84	33.93	988	195	4	62.20
AE	80.54	45.04	2381	172.39	4.32	-0.22	13.79	34.92	968	466	17	42.60
AE	84.12	42.70	2427	353.37	0.67	-3.09	13.22	27.14	1326	243	3	91.52
AE	81.16	44.46	1917	242.22	1.63	0.80	11.83	28.98	1063	376	14	43.63
AE	81.33	42.57	2422	45.22	13.70	0.63	13.10	31.26	1032	276	5	76.02
AE	82.39	43.60	780	334.65	0.16	8.92	12.78	27.47	1227	196	7	47.34
AE	81.07	43.44	2427	106.01	7.12	-0.20	11.81	29.59	1003	404	10	60.06
AE	83.47	43.70	1863	299.43	5.65	1.27	11.75	27.52	1155	277	5	67.89
AE	87.12	43.29	2469	62.80	6.84	1.00	12.78	32.84	981	272	3	85.85
AE	83.26	44.40	2171	293.53	4.18	-1.16	11.29	27.27	1111	308	6	66.86
AE	80.49	41.26	1169	160.35	0.23	10.35	15.13	31.91	1172	86	2	69.43
AE	76.21	39.46	1252	161.57	0.05	11.64	13.09	30.38	1112	85	2	53.40
AE	80.40	41.49	1484	185.33	4.25	9.10	14.64	30.70	1172	114	3	72.47
AG	76.91	40.18	1620	180.97	0.46	9.45	13.93	31.29	1125	102	2	61.30
AG	89.02	45.05	773	214.13	1.97	6.94	13.56	26.07	1418	153	6	36.97
AG	91.66	43.79	1651	175.60	4.03	4.37	11.96	26.63	1237	117	2	75.06
AG	82.17	43.61	806	200.39	4.02	8.78	12.93	27.58	1236	210	9	41.65
AG	75.48	37.23	3832	325.42	7.82	0.20	14.50	34.77	1013	64	0	71.99
AG	91.38	44.97	1186	120.70	1.73	5.87	12.61	27.11	1270	99	2	72.17

AG	86.01	43.02	3334	12.27	4.51	-5.52	12.21	29.85	1064	317	2	93.86
AG	85.59	46.80	1364	183.81	2.09	3.76	11.70	27.79	1157	258	8	50.90
AG	89.94	43.10	925	169.96	1.64	9.27	14.26	28.12	1339	69	1	66.29
AG	82.58	45.58	1571	351.66	3.04	3.40	12.73	29.19	1140	293	9	51.56
AG	91.39	45.06	1314	114.78	1.99	5.24	12.38	27.22	1246	109	2	76.52
AG	90.37	45.55	1230	247.01	0.56	4.29	12.57	27.32	1257	133	5	50.46
AG	81.14	44.78	1893	54.65	4.95	1.96	12.83	30.63	1071	358	13	45.95
AG	82.50	43.40	1015	226.10	6.80	7.90	12.58	26.81	1251	210	7	51.82
AG	82.35	43.69	1404	219.43	3.49	5.55	12.76	27.86	1202	266	8	52.57
AG	93.85	43.13	1519	283.84	1.10	5.82	13.02	29.32	1173	107	2	76.61
AG	94.81	43.32	1488	355.07	1.80	5.11	12.62	28.48	1196	73	1	93.73
AG	89.12	44.75	499	218.29	0.19	7.49	13.63	24.42	1555	135	5	35.35

Note: AE: *A. euchroma*; AG: *A. guttata*; Long: Longitude; Lat: Latitude; ELE: Elevation; ASP: Aspect; SLP: Slope; bio1: Annual mean temperature; bio2: Mean diurnal range; bio3: Isothermality; bio4: Temperature seasonality; bio12: Annual precipitation; bio14: Precipitation of driest month; bio15: Precipitation seasonality.

Table S7. Soil data at sampling points.

Species	Long	Lat	AP	AK	AN	SOM	t_ph	t_clay	t_sand
AE	86.22	44.30	7.00	108.78	64.10	202123	8.00	18	34
AE	90.52	43.56	4.85	138.00	263.76	202123	7.50	20	35
AE	82.42	42.37	5.94	173.76	97.96	202123	6.30	20	46
AE	83.94	43.17	3.85	107.93	260.74	202123	5.70	6	56
AE	75.08	37.83	4.31	98.80	88.46	302131	6.30	20	46
AE	74.58	39.91	3.76	85.13	157.45	202123	5.70	6	56
AE	80.54	45.04	4.00	137.00	228.26	202123	7.50	20	35
AE	84.12	42.70	2.14	51.98	103.74	202123	6.30	20	46
AE	81.16	44.46	4.43	113.37	275.45	202123	7.50	20	35
AE	81.33	42.57	3.85	107.93	260.74	202123	5.70	6	56
AE	82.39	43.60	6.01	155.60	105.11	202123	7.90	22	35
AE	81.07	43.44	4.00	137.00	228.26	202123	7.50	20	35
AE	83.47	43.70	4.43	113.37	275.45	202123	7.50	20	35
AE	87.12	43.29	5.87	135.00	167.57	202123	7.50	20	35
AE	83.26	44.40	9.00	139.68	134.11	202123	6.60	21	25
AE	80.49	41.26	1.35	59.69	81.47	203223	6.30	47	10
AE	76.21	39.46	5.50	59.69	72.28	203223	6.30	47	10
AE	80.40	41.49	3.05	269.00	103.23	303223	7.90	22	34
AG	76.91	40.18	7.00	303.44	43.00	102113	7.60	3	92
AG	89.02	45.05	4.29	98.45	34.21	202213	5.70	5	89
AG	91.66	43.79	4.37	112.63	47.21	202123	8.00	26	29
AG	82.17	43.61	5.97	187.81	34.67	102113	8.10	21	41
AG	75.48	37.23	4.31	98.80	88.46	302131	6.30	20	46
AG	91.38	44.97	5.58	108.06	25.08	303222	8.10	26	45

AG	86.01	43.02	3.78	110.07	282.38	202123	5.70	6	56
AG	85.59	46.80	4.55	105.52	26.49	202223	8.10	26	45
AG	89.94	43.10	3.05	269.00	103.23	303213	7.90	22	34
AG	82.58	45.58	4.37	112.63	47.21	202123	8.00	26	29
AG	91.39	45.06	5.58	108.06	25.08	303212	8.10	26	45
AG	90.37	45.55	4.29	139.94	48.27	303213	7.90	22	34
AG	81.14	44.78	4.82	122.40	56.21	202123	7.30	21	37
AG	82.50	43.40	5.97	187.81	34.67	102113	8.10	21	41
AG	82.35	43.69	4.37	112.63	47.21	202123	7.30	22	36
AG	93.85	43.13	5.58	108.06	25.08	303222	8.10	26	45
AG	94.81	43.32	4.55	105.52	26.49	202223	8.10	26	45
AG	89.12	44.75	4.29	98.45	34.21	202213	5.70	5	89

Note: AE: *A. euchroma*; AG: *A. guttata*; Long: Longitude; Lat: Latitude; AP: Available phosphorus content; AK: Available potassium content; AN: Available nitrogen content; SOM: Soil organic matter content; t_ph: Soil pH; t_clay: Soil clay fraction; t_sand: Sand percentage

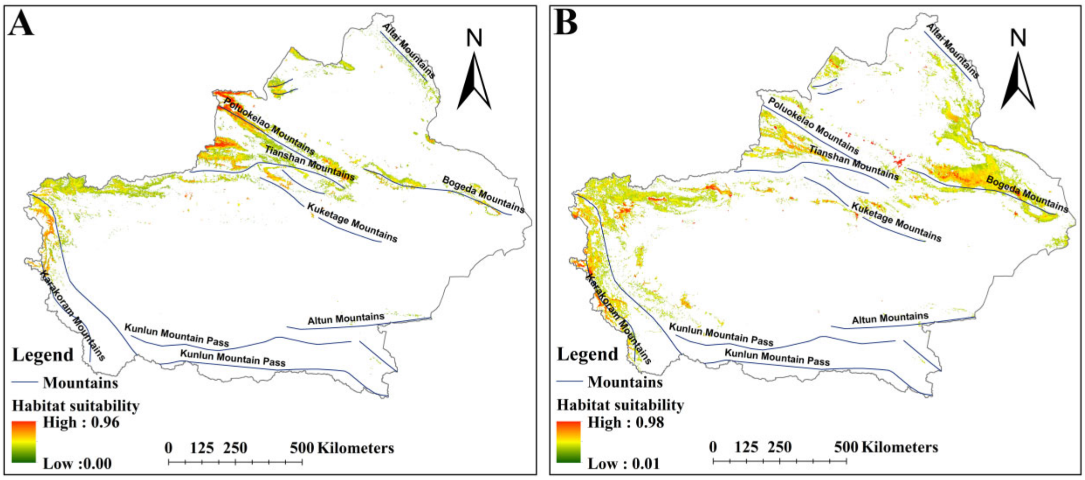

Figure S1. Habitat suitability distribution map of two medicinal plants under the current scenario. (A) *A. euchroma*, (B) *A. guttata*.

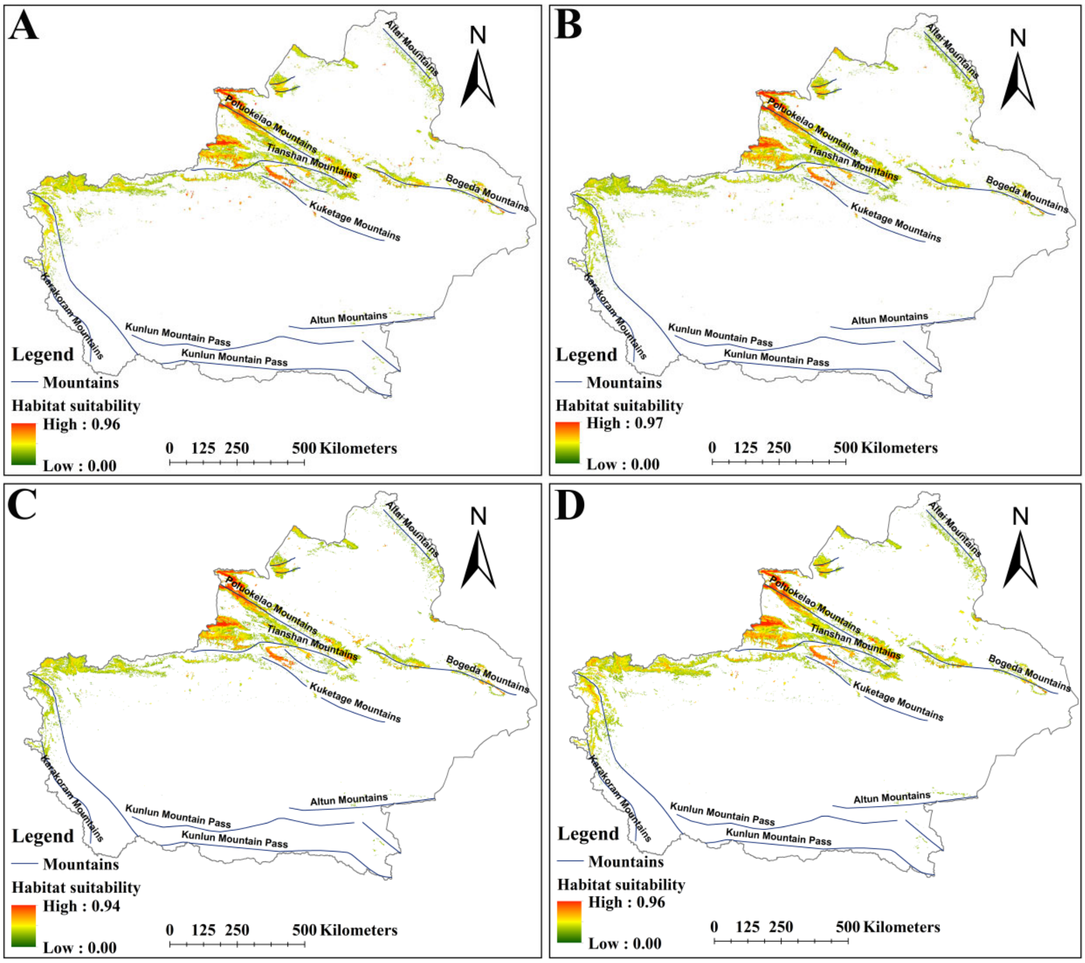

Figure S2. Habitat suitability distribution map of *A. euchroma* under future climate scenarios. (A) ssp126-2050s, (B) ssp585-2050s, (C) ssp126-2090s, (D) ssp585-2090s.

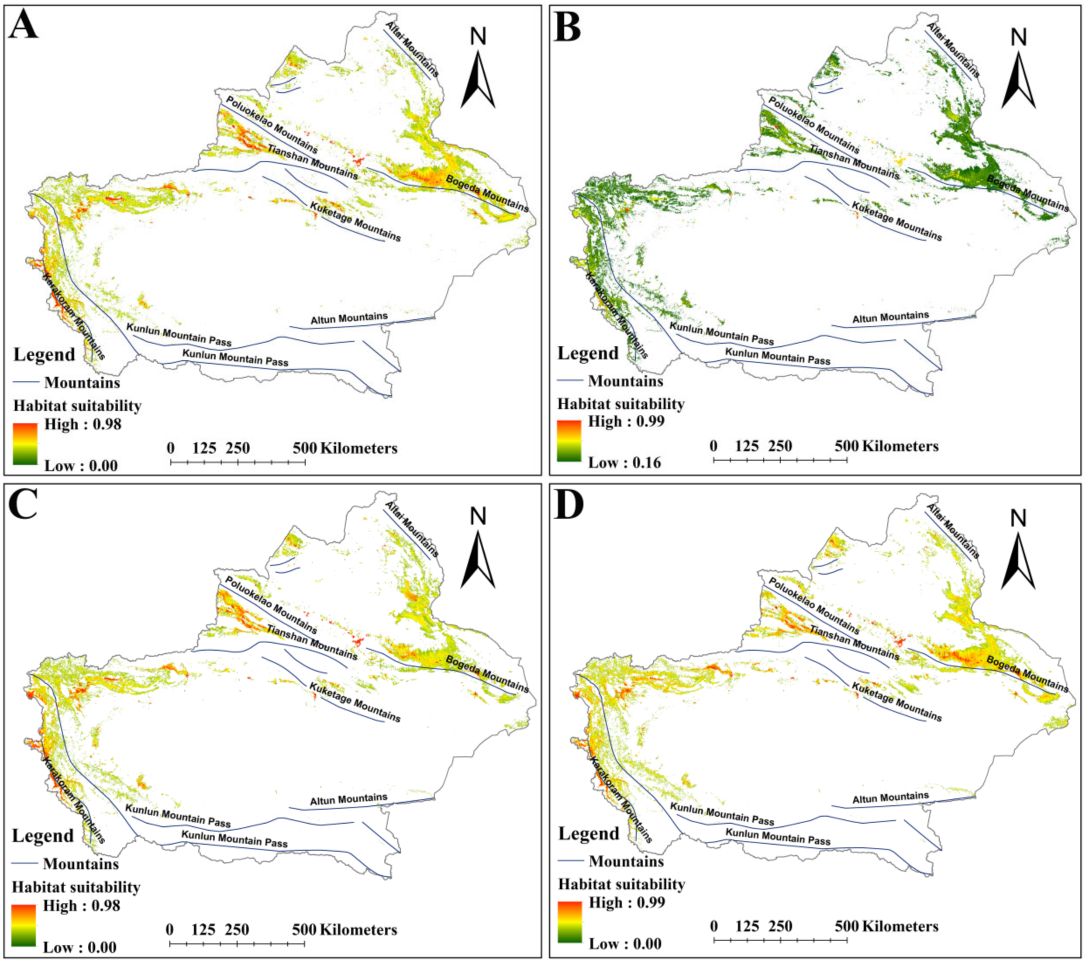

Figure S3. Habitat suitability distribution map of *A. guttata* under future climate scenarios. (A) ssp126-2050s, (B) ssp585-2050s, (C) ssp126-2090s, (D) ssp585-2090s.

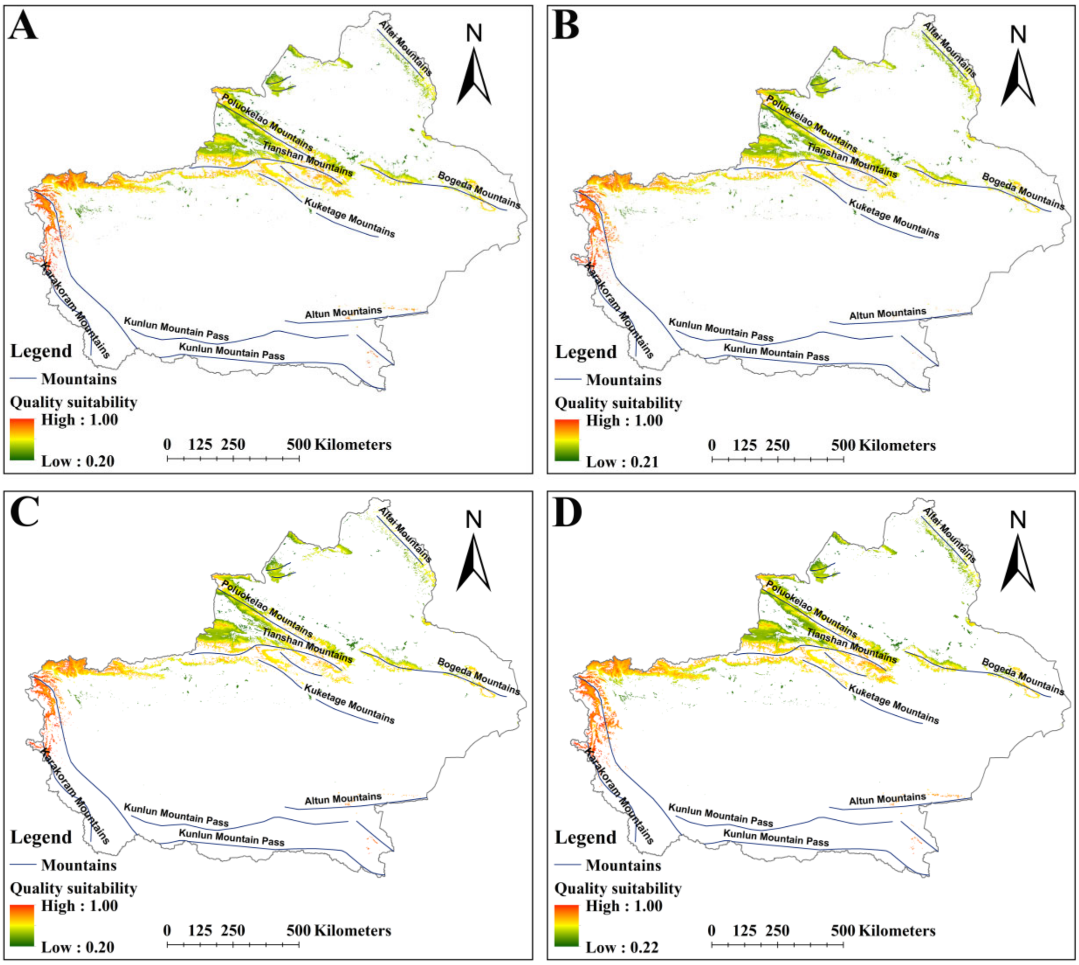

Figure S4. Quality suitability distribution map of *A. euchroma* under future climate scenarios. (A) ssp126-2050s, (B) ssp585-2050s, (C) ssp126-2090s, (D) ssp585-2090s.

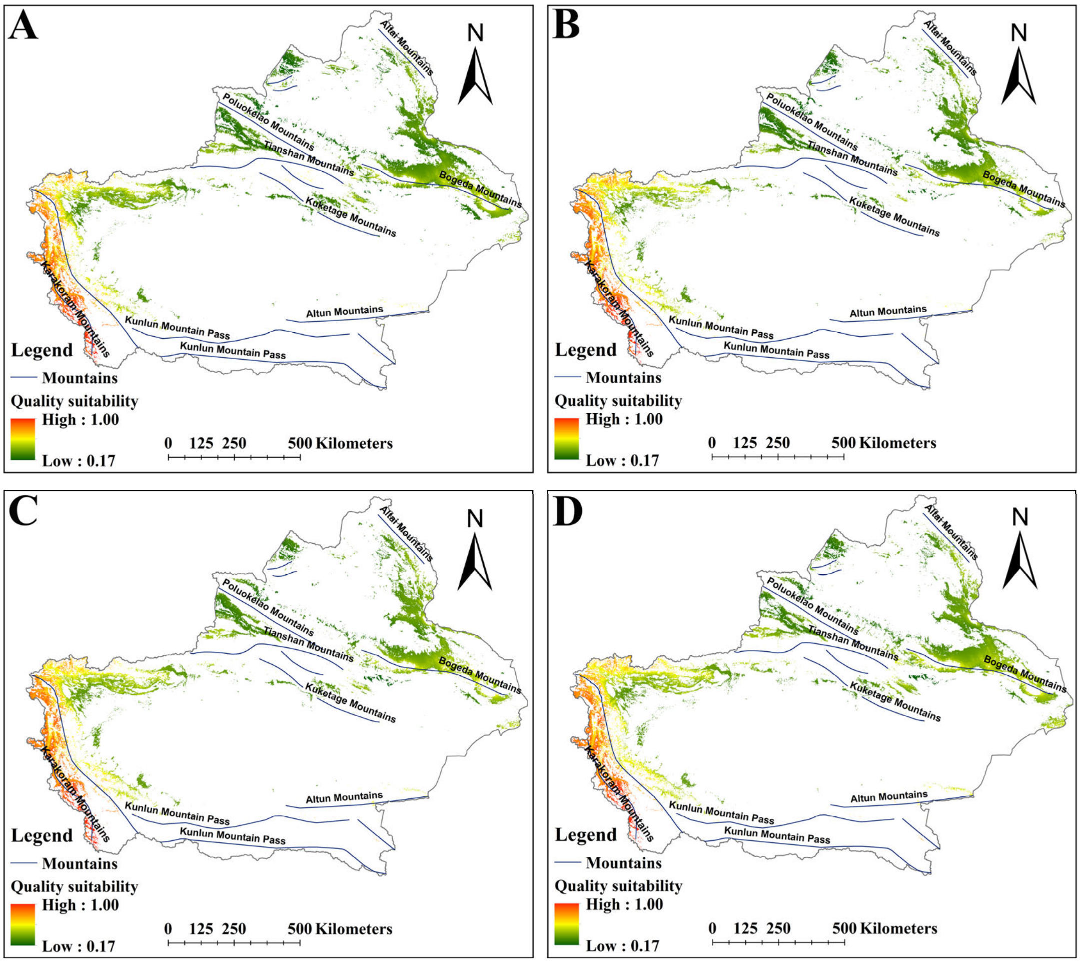

Figure S5. Quality suitability distribution map of *A. guttata* under future climate scenarios. (A) ssp126-2050s, (B) ssp585-2050s, (C) ssp126-2090s, (D) ssp585-2090s.
